# 3D-printed nanohydroxyapatite/methylacrylylated silk fibroin scaffold for repairing rat skull defects

**DOI:** 10.1186/s13036-024-00416-5

**Published:** 2024-03-21

**Authors:** Wu Huiwen, Liang Shuai, Xie Jia, Deng Shihao, Wei Kun, Yang Runhuai, Qian Haisheng, Li Jun

**Affiliations:** 1 Department of Orthopedics, The Second Affiliated Hospital of Anhui Medical University, Hefei, 230601 China; 2 Institute of Orthopedics, Research Center for Translational Medicine, The Second Affiliated Hospital of Anhui Medical University, Hefei, 230601 China; 3 School of Biomedical Engineering, Anhui Provincial Institute of Translational Medicine, Anhui Medical University, Hefei, 230032 People’s Republic of China

**Keywords:** Nanohydroxyapatite, Silk fibroin, Bone defect, 3D printing

## Abstract

The repair of bone defects remains a major challenge in the clinic, and treatment requires bone grafts or bone replacement materials. Existing biomaterials have many limitations and cannot meet the various needs of clinical applications. To treat bone defects, we constructed a nanohydroxyapatite (nHA)/methylacrylylated silk fibroin (MASF) composite biological scaffold using photocurable 3D printing technology. In this study, scanning electron microscopy (SEM) was used to detect the changes in the morphological structure of the composite scaffold with different contents of nanohydroxyapatite, and FTIR was used to detect the functional groups and chemical bonds in the composite scaffold to determine the specific components of the scaffold. In in vitro experiments, bone marrow mesenchymal stem cells from SD rats were cocultured with scaffolds soaking solution, and the cytotoxicity, cell proliferation, Western blot analysis, Quantitative real-time PCR analysis, bone alkaline phosphatase activity and alizarin red staining of scaffolds were detected to determine the biocompatibility of scaffolds and the effect of promoting proliferation and osteogenesis of bone marrow mesenchymal stem cells in vitro. In the in vivo experiment, the skull defect was constructed by adult SD rats, and the scaffold was implanted into the skull defect site. After 4 weeks and 8 weeks of culture, the specific osteogenic effect of the scaffold in the skull defect site was detected by animal micro-CT, hematoxylin and eosin (HE) staining and Masson's staining. Through the analysis of the morphological structure of the scaffold, we found that the frame supported good retention of the lamellar structure of silk fibroin, when mixed with nHA, the surface of the stent was rougher, the cell contact area increased, and cell adhesion and lamellar microstructure for cell migration and proliferation of the microenvironment provided a better space. FTIR results showed that the scaffold completely retained the β -folded structure of silk fibroin, and the scaffold composite was present without obvious impurities. The staining results of live/dead cells showed that the constructed scaffolds had no significant cytotoxicity, and thw CCK-8 assay also showed that the constructed scaffolds had good biocompatibility. The results of osteogenic induction showed that the scaffold had good osteogenic induction ability. Moreover, the results also showed that the scaffold with a MASF: nHA ratio of 1: 0.5 (SFH) showed better osteogenic ability. The micro-CT and bone histometric results were consistent with the in vitro results after stent implantation, and there was more bone formation at the bone defect site in the SFH group.This research used photocurable 3D printing technology to successfully build an osteogenesis bracket. The results show that the constructed nHA/MASF biological composite material, has good biocompatibility and good osteogenesis function. At the same time, in the microenvironment, the material can also promote bone defect repair and can potentially be used as a bone defect filling material for bone regeneration applications.

**Supplementary Information:**

The online version contains supplementary material available at 10.1186/s13036-024-00416-5.

## Introduction

Trauma, tumor, infection and congenital malformation are common factors resulting in bone defects, and the repair of bone defects has always been an important problem for orthopedic clinicians to solve [1–3]. It is estimated that more than 1.5 million patients are involved with bone defects in the United States each year, and more than 2 million bone grafts are implanted worldwide each year, more than a quarter of which are performed in the United States [4]. The main method of bone defect repair is bone graft, which includes autografts and allografts. To date, the most common method for treating bone deficiencies is autologous bone grafting [5, 6], but there are many limitations in terms of treatment method [7]. Major complications such as infection, protracted wound drainage, hematomas, and reoperation were reported at an 8.6% [8]. In recent years, the development of a variety of biomaterials for autologous bone transplantation in bone tissue engineering has emerged. Hydroxyapatite (HA), β -tricalcium phosphate (β-TCP) and their composites are promising biomaterials widely used in orthopedic reconstructive surgery due to their similar composition to normal bone [9–11]. These biomaterials have been employed in the treatment of clinical bone defects.

Most HA in the human body exists in a solid or ionic state and is dynamically involved in the metabolic processes of bone resorption and bone reconstruction [12]. Studies have shown the porous ceramic materials prepared by HA can effectively promote the formation of new bone in rabbit bone defects, while the osteogenic effect of traditional ceramics is much lower than that of porous scaffolds due to their dense structure [13]. Other studies have shown that hydroxyapatite provides a surface that facilitates bone cell attachment and proliferation, known as bone conductivity [14–16]. In recent studies, many scaffolders have been prepared using nano-HA, because HA has particular affinity for a number of adhesive proteins and is directly involved in the process of bone cell differentiation and mineralization [17]. Nevertheless, the limited pace at which it is absorbed may hinder its breakdown within the body and postpone the initiation of new bone development. Moreover, the innate rigidity, fragility, and absence of pliability all restrict its usage in clinical settings [18, 19]. The field of engineering biomaterials, namely inorganic–organic composites, has gained significant attention and emphasis in recent decades due to its ability to replicate the structure and chemistry of natural tissues. A novel HA-chitosan substance has emerged. The presence of pores, ability to break down naturally, and ability to kill germs make chitosan a desirable material for bone tissue creation. Nevertheless, the mechanical characteristics of chitosan-based composites are inadequate and require enhancement [20, 21]. The HA-polycaprolactone material is suitable for bone regeneration due to its favorable bioresorbability and cost-effectiveness. Nevertheless, its efficacy as a bioactive substance is limited due to the weak affinity it has with bone tissue [22].

Silk fibroin(SF) is a sustainable polymer from nature, which has a structure of twelve repetitive hydrophobic domains separated by short hydrophilic linker sequences [23]. It has been widely developed for tissue engineering and regenerative medicine, bioelectronics, and biooptics [24, 25]. The hydrophobic regions arrange themselves into crystalline and semi-crystalline b-sheet structures, resulting in a distinctive blend of stability, strength, and toughness [26]. What is more. The inherent chemical and physical composition of SF enables its modification using diverse crosslinking techniques [27]. These methods, such as ultrasonication, shearing, surfactants, the use of anionic agents and non-enzymatic or enzymatic crosslinker, change the structure of SF and improve its mechanical stability [28–30]. A glycidyl-methacrylate-modified SF solution was developed, Methacryloylated silk fibroin (MASF), which can be chemically crosslinked within a few minutes using light, has stable physicochemical properties, is biocompatible and is highly scalable, offering a wide range of viscoelastic properties suitable for different applications [31]. Reprocessing of silk fibroin in aqueous solution gives it versatility and subsequently forms different material forms, including fibers, films, sponges and hydrogels [32, 33]. Substantial research has demonstrated the great potential of SF in bone repair, but the silk fibers lack sufficient calcium ion binding sites, resulting in poor mineralization ability and further optimization [34]. Therefore, the combination of nHA and MASF as scaffold materials can fully leverage their advantages for bone conduction and bone induction and maximize the physical and chemical properties of scaffold materials.

**Fig. 1:**
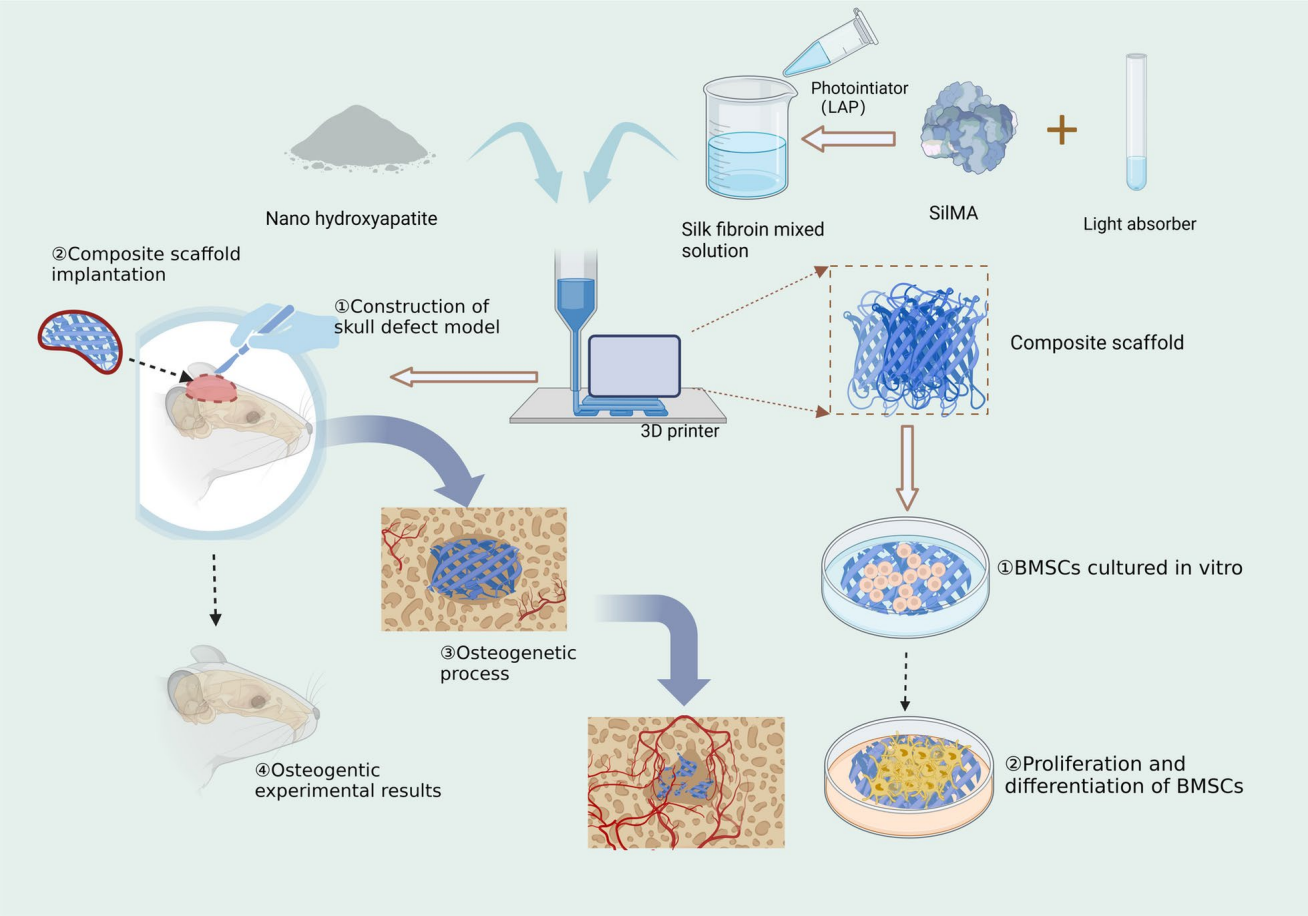
The fabrication of photocured 3D printed nHA/MASF composite scaffolds and its planar schematic for promoting bone regeneration in vivo and in vitro

Herein, the nHA/MASF composite scaffolds were prepared by photocured 3D printing in an inorganic‒organic combination mode in this study (Fig. 1). The two materials complement each other, and the combination of the two improves bone inductance and bone conductivity. To confirm the osteogenic efficacy of the scaffolds and the exact effect on bone defect repair, in vitro and in vivo experiments were performed to explore the osteogenic differences of scaffolds with different mixing ratios. Ultimately, the 0.5:1 nHA/MASF composite scaffold showed excellent bone regeneration ability. Hence, the proposed 3D printed nHA/MASF composite scaffold has a promising application in the repair of bone defects.

## Materials and methods

### Preparation of 3D printing solution

One gram of MASF (Engineering for Life, EFL-SiLMA-001) was placed in a centrifuge tube, and 0.05% (w/v) ultraviolet absorber water soluble (UV-326, Milanchemical, 3896–11-5) was added to the centrifuge tube. Nanohydroxyapatite (Macklin, China, H875578) was weighed to achieve mass ratios of MASF: nHA of = 1:0, 1:0.5, and 1:1 and added to the centrifuge tube. The centrifuge tube was filled with photoinitiator standard solution composed of: Lithium Phenyl (2,4,6-trimethylbenzoyl) phosphinate (EFL-LAP, Engineering for Life, EFL-LAP-1). The solution was dissolved at ambient temperature for 0.5–1 h, during which the solution was stirred/shaken several times (to avoid severe ultrasound, high temperature and strong shear). The MASF solution was sterilized with a sterile 0.22 μm needle filter. The filtered solution was stored at low temperature about 4 °C.

### Fabrication of scaffolds

To produce the scaffold, a digital light processing bioprinting (EFL Light Curing Biological 3D Printer, China, BP8600) with high accuracy about 25 μm was used.The solution prepared above was poured into the printer's feed tank, and the material was printed according to the following parameters: Setting the print height to 100 μm, light intensity to10 mW/cm^2^, and single-layer exposure time to15 s. This is the optimal parameter that has been tested and determined. Printing parameters have an impact on printing accuracy. Too much light intensity or too long a printing time will affect the molding accuracy of the printed scaffolds. The smaller the print height, the higher the molding accuracy. If the light intensity is too low or the printing time is too short, then the printed scaffolds are difficult to be shaped. After printing, the solution was removed. Three scaffolds with different proportions (Mass ratios of MASF: nHA of = 1:0, 1:0.5, and 1:1) were prepared with 5-mm diameter and 1-mm height, soaked and cleaned in PBS solution for several times, and then soaked in PBS for preservation. Design of a digital model of the 5-mm diameter and 1-mm height 3D bracket using the computer-aided design software SolidWorks. The layer resolution ranged from 400 to 600 μm for a high fidelity of the 3D-printed scaffolds structure.

### Characterization of scaffolds

The apparent forms of the nHA/MASF composite scaffold were characterized by scanning electron microscopy (SEM, Sigma 300, ZEISS, Germany). The chemical components of the scaffolds were characterized using Fourier transform infrared spectroscopy. Compression measurements of the scaffolds were performed along the print axis (z-axis) with a universal testing machine (Shimazu, AG-2000A, Japan) using displacement control (4 mm/min) at ambient conditions and at least three specimens were measured for each sample for the above test.

### Cell proliferation assay

The influence of the scaffolds on cell viability and proliferative activity was measured. Bone mesenchymal stem cells (BMSCs) were cultured in α-MEM medium (Gibco, USA) containing 10% fetal bovine serum (FBS) (Gibco, USA) and 1% penicillin/streptomycin (Gibco, USA) at 37 °C and 5% CO2. The culture medium was replaced every 2 days, and once the cells achieved 80% confluency, they were treated with trypsin (Gibco, USA). Cells were utilized from the third to fifth generation. The cell inoculation involved 5.0 × 10^4^ cells/cm^2^. In order to prepare scaffold extracts,in accordance with the international standard ISO 10993–12: 2021 ' Biological evaluation of medical devices—Part 12: Sample preparation and reference samples ',three scaffolds with different proportions (Mass ratios of MASF: nHA of = 1:0, 1:0.5, and 1:1) were cultured in 1 ml by the above α-MEM medium at 37 °C and 5% CO2 for 24 h. The effect of the extracts on BMSCs was tested using a medium containing 10% of the extracts. We designed four groups: the 1:0 group (SF group), 1:0.5 group (SFH group), 1:1 group (SFHH group) and blank group without extract (N group). Cells were seeded into 24-well tissue culture plates at a density of 5 × 10^4^ cells/cm^2^. The cell viability was analyzed by a live/dead cell staining experiment (Beyotime, China). After a 24-h culture, 100 µl staining working solution was added to each well and incubated at 37 °C for 15 min. Live cells (green fluorescence, 490 nm) and dead cells (red fluorescence, 545 nm) were simultaneously detected under a fluorescence microscope.

### Cell counting kit-8 (CCK-8) analyze cytotoxicity

The cell seeding and culture protocol was the same as detailed above. Briefly, 0.5 mL of fresh DMEM containing 10% CCK-8 solution (Biosharp, United States) was added to each well. After 2 h, 100 µL of the mixed medium was transferred to a 96-well plate. The solution absorbance was measured at a wavelength of 450 nm with a microplate reader (Thermo, United States).

### Alkaline phosphatase activity

To determine the effect of the scaffold on the alkaline phosphatase (ALP) activity of BMSCs, osteogenic differentiation medium containing scaffold immersion solution was used to induce differentiation, and groups were treated as described previously. Medium for rat BMSCs osteogenic differentiation (Cyagen, China) was prepared as the product manual. Cultivation of medium for rat BMSCs osteogenic differentiation containing 10% V/V scaffold soaking solution. After 1 week and 2 weeks of osteogenic induction, the cells were digested, centrifuged and collected into centrifuge tubes. Ice lysis cells were processed with 100 μL RIPA cell lysis solution (without phosphatase inhibitors and protease inhibitors) for 30 min and centrifuged at 13,500 r in a 4 °C high speed centrifuge for 30 min, and then collected the supernatant. The ALP activity was detected by an ALP kit (Beyotime, China). A BCA protein detection kit (Beyotime, China) was used to measure the total protein content in the extracted samples and standardized alkaline phosphatase activity by the total protein content.

### Alizarin red S staining

Alizarin red S staining was used to detect the mineralized nodules of BMSCs in SD rats. The cells were inoculated at a density of 5 × 10^4^/ml on a 24-well plate and incubated for 24 h, then medium for rat BMSCs osteogenic differentiation (Cyagen,Chian) containing 10% V/V scaffold immersion solution was used. Then BMSCs cultured in medium containing 10% V/V scaffold immersion solution for 14 and 21 days, and the groups were treated as described previously. At a predetermined time, the cells were fixed with a 4% paraformaldehyde solution. The immobilized cells were further washed in pure water to remove any salt residues, added to a 2% (WT/V) Alizarin red S (ARS, Solebo, China) solution and incubated at room temperature for 30 min. After the samples were dried, fluorescence microscopy was used to collect images. ImageJ was used to quantify the images taken, and detection was carried out in triplicate.

### Western blot analysis

The protein expression of SD rat BMSCs was detected by Western Blotting. After 24 h, the medium was replaced with normal osteogenic induction medium and osteogenic induction medium containing 10% of different scaffold extracts. After culturing for 14 days, cell precipitation was collected; 100 µl of RIPAcell lysate (Beyotime, China) containing 1 mM PMSF, (Beyotime, China) was added to each 6-well plate, lysed, centrifuged, and collected the supernatant. Total cellular protein was quantified using a BCA protein assay kit. Then the samples were subjected to SDS electrophoresis and transferred to a polyvinylidene fluoride membrane. The membranes were first blocked with 5% skim milk for 1 h, and then incubated at 4 °C overnight with primary antibodies as follows: RUNX2 (AF5186, Affinity, 1:500), OCN (DF12303, Affinity, 1:500), ALP (DF6225, Affinity, 1:500), and Collagen I (AF7001, Affinity, 1:500). After being washed with TBST three times, the membranes were incubated with HRP-conjugated secondary antibodies (1:10,000). The antigen–antibody complex was visualized. Signal intensities were quantified using ImageJ software.

### Quantitative real-time PCR analysis

The cells were seeded at a density in tissue culture plates and grouped as before. After 24 h, the osteogenic induction protocol was the same as before. After culturing for 14 days, total RNA was extracted from cells according to the manufacturer’s instructions. Complementary DNA was synthesized from total RNA using the PrimeScript RT Reagent Kit. After reverse transcription, real-time PCR was performed using a SYBR Green qPCR kit (Servicebio, China) and an ABI Step One Plus Real-Time PCR System. Each sample was analyzed in triplicate, and β-actin was used as a reference. The primer sequences used are described in Table 1.

**Table 1:** Information on the primers designed for each osteogenic protein genes

Gene	Amplicon Size(bp)	Forward primer(5' → 3')	Reverse primer(5' → 3')
Mo-β-actin	120	AGTGTGACGTTGACATCCGT	TGCTAGGAGCCAGAGCAGTA
Mo-RUNX2	130	AAATTGCAGGCTTCGTGGTT	AATGGCTGTATGGTGAGGCT
Mo-OPN	182	CCTGACCCATCTCAGAAGCA	TCCACAGAATCCTCGCTCTC
Mo-ALP	185	GTATGGGCGTCTCCACAGTA	CTTCACGCCACACAAGTAGG
Mo-Col1a1	88	AACTGGTACATCAGCCCGAA	TTCCGTACTCGAACGGGAAT
Mo-OCN	156	AGTGTGAGCTTAACCCTGCT	GAGGATCAAGTCCCGGAGAG

### Establishment of the skull defect model

Female SD rats were used as the object of the study on osteogenic performance in vivo. Forty-eight SD rats were randomly divided into 4 groups with 12 rats in each group, and one group was the sham operation group. All the experimental procedures were carried out on animals with the formal approval of the Ethics and Animal Research Committee of Anhui Medical University, China (NO. YX2022-128). Clean environment feeding, free eating, drinking water, regular environmental disinfection. The rats were weighed by electronic balance, anesthetized by chloral hydrate, and fixed in the prone position on the operating table. The scalp hair of rats was shaved to expose the skin, the skin was disinfected with an iodine cotton ball, the sterile hole towel was covered, and the operation was carried out under strict aseptic conditions. First, the median line of the skull was determined, and then a skin incision of approximately 2 cm in length was made along its length. The skin and subcutaneous tissue were cut to the periosteum layer in turn, and the frontal bone and skull were fully exposed. The skull was drilled vertically at low speed using a surgical electric hollow trephine, approximately 4 mm in diameter. Normal saline was added regularly during drilling to avoid damage to brain tissue and blood vessels caused by the high temperature of the drill bit and friction. When a slight break was felt with the drill, the skull fraction along the circular hole was removed with tweezers. The nHA/MASF scaffold was implanted and sutured layer by layer. The incision was disinfected with iodophor for 3 consecutive days after the operation, and 200,000 U penicillin was injected subcutaneously once a day. The room temperature was maintained at (25 ± 2) °C.

### Radiographic evaluation

At 4 and 8 weeks after stent implantation, 3 SD rats from each group were sacrificed by cervical dislocation at each time point, and the skulls were separated. The samples were fixed in paraformaldehyde solution and scanned using a micro-CT system (Skyscan1276, Bruker, Germany) to evaluate the new bone around the defect area. A cylindrical area of interest (ROI) with an appropriate diameter and depth was selected in the bone defect area and reconstructed by a CT analyzer. Bone volume/total volume (BV/TV), bone trabecular number (Tb.N) and bone surface area (BS) were calculated in the CTAn procedure (Skyscan, Germany) to evaluate new bone tissue.

### Histological analysis

For analysis of bone histology, craniums were fixed in paraformaldehyde, and subsequently decalcified for four weeks before embedding in paraffin, and thick sections were used for hematoxylin and eosin (HE) staining and Masson’s trichrome staining.

### Statistical analysis

Statistical analysis was performed using SPSS 19 (IBM, Armonk, IL, USA). All results are expressed as the mean ± SD. The results were analyzed by independent T test and one-way ANOVA. *P* < 0.05 was statistically significant. All quantization was performed on high-resolution images using ImageJ, followed by statistical analysis and mapping of the data using GraphPad Prism 8.0.1.

## Results

### Morphology and functional group analysis of the scaffolds

**Fig. 2:**
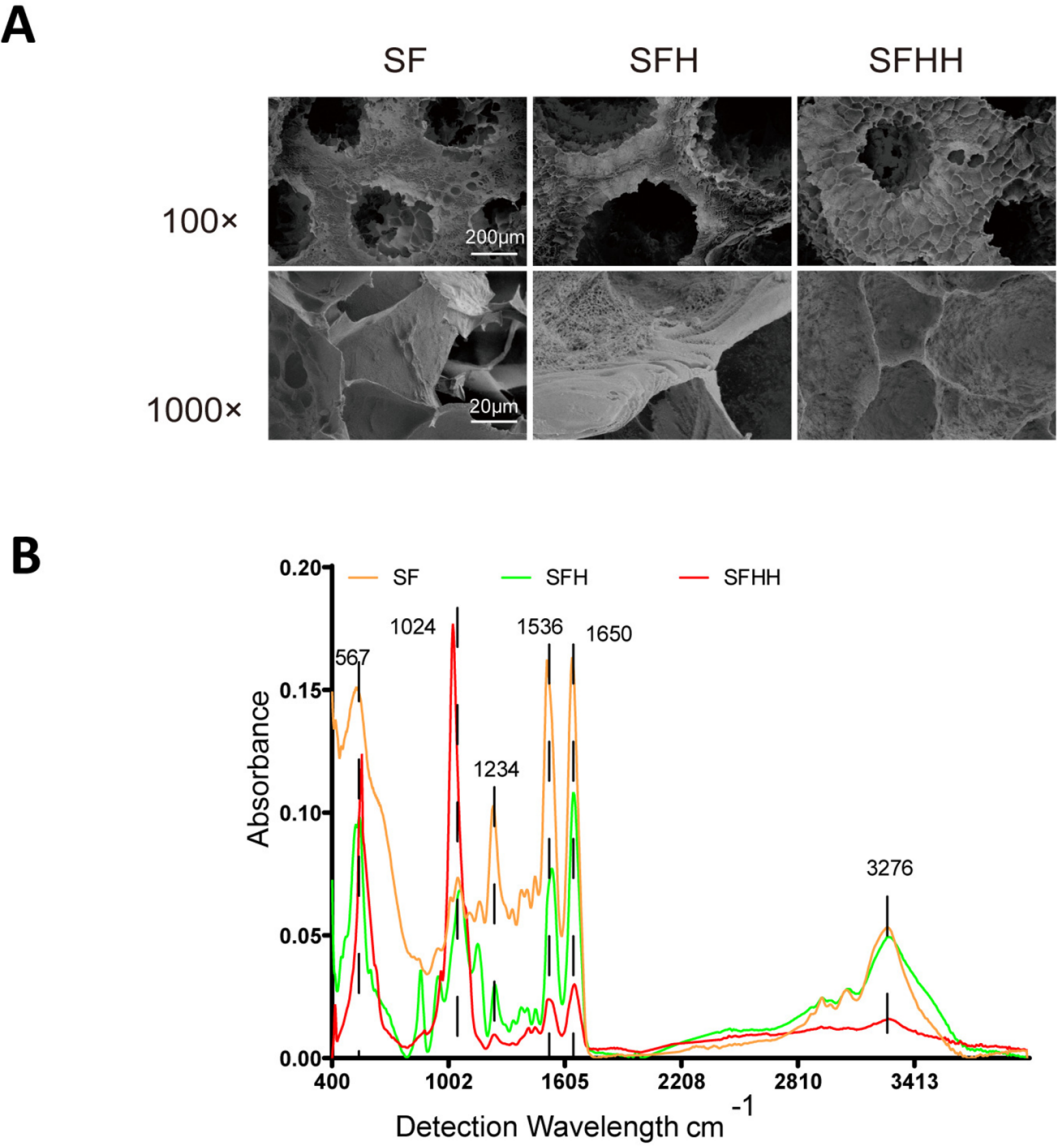
Structural and characterizations of nHA/MASF scaffolds. **A** SEM images of nHA/MASF scaffolds. **B** FTIR spectra of scales of the nHA/MASF scaffolds. SF: nHA/MASF = 1:0, SFH: nHA/MASF = 1:05, SFHH: nHA/MASF = 1:1

The scaffolds were fabricated with a grid structure and printed layer by layer to ensure a 3D porous space structure simulating trabecular bone (Fig. 2). Nanohydroxyapatite did not change the macrostructure of MASF, and all scaffolds retained the flake structure of MASF. The surface morphology of the scaffold changed with increasing nanohydroxyapatite content. With the increase of nano-hydroxyapatite content, the surface of the scaffold became rough and micropores on the surface increased, and the surface area increased. However, further addition of nanohydroxyapatite made the void on the surface of the scaffold larger. At high magnification, it was observed that the surface of the scaffold containing hydroxyapatite showed uniform protrusions, which showed a more compact protrusion structure than that of the SF and SFH. The SFH group has a stent appearance that is better suited to roughness and has more appropriately sized pores.

FTIR spectroscopy was used to evaluate the secondary conformation of silk fibroin protein and the effect of adding hydroxyapatite to the scaffold. FTIR is very sensitive to the conformational modification of scaffolds. Different related amide groups in proteins exhibit specific absorption bands in different vibration regions. FTIR is very sensitive to the conformational modification of scaffolds. Different related amide groups in proteins exhibit specific absorption bands in different vibration regions. The mostly significant infrared band for silk protein analysis is the amide band: the peak of the amide I region near 1650 cm^−1^ (C = O stretching), the peak of the amide II region near 1536 cm^−1^ (NH bending), the peak of the amide III region near 1234 cm^−1^ (C-N stretching), the peak of acidamide a near 3276 cm^−1^ (hydrogen bonding) and the peak of amide V near 567 cm^−1^. The amino acid conformation of silk fibroin in silkworms is characterized by β -folded absorption peaks at 1630, 1530 and 1240 cm^−1^; regular helix absorption peaks at 1650 or 1645, 1550 and 1230 cm^−1^; and α -helical absorption peaks at 1655 cm^−1^. The peak near 3300 cm^−1^ appears in response to fluctuations in the hydrogen bond. Therefore, the β -folding of the scaffold and the conformation of the relevant amide groups are not changed. The peak near 1024 cm^−1^ is attributed to PO bending of the phosphate group (PO_4_^3−^), indicating the presence of nHA (Fig. 2). The scaffolds of SFHH because of the increase with the mass ratio of nHA and the relative decrease with the mass ratio of MASF, resulting in an increase of the absorption peak near 1024 cm^−1^ and a decrease in the absorption peak which represents the β-folding (Fig. 2).

### Cell proliferation assay

**Fig. 3:**
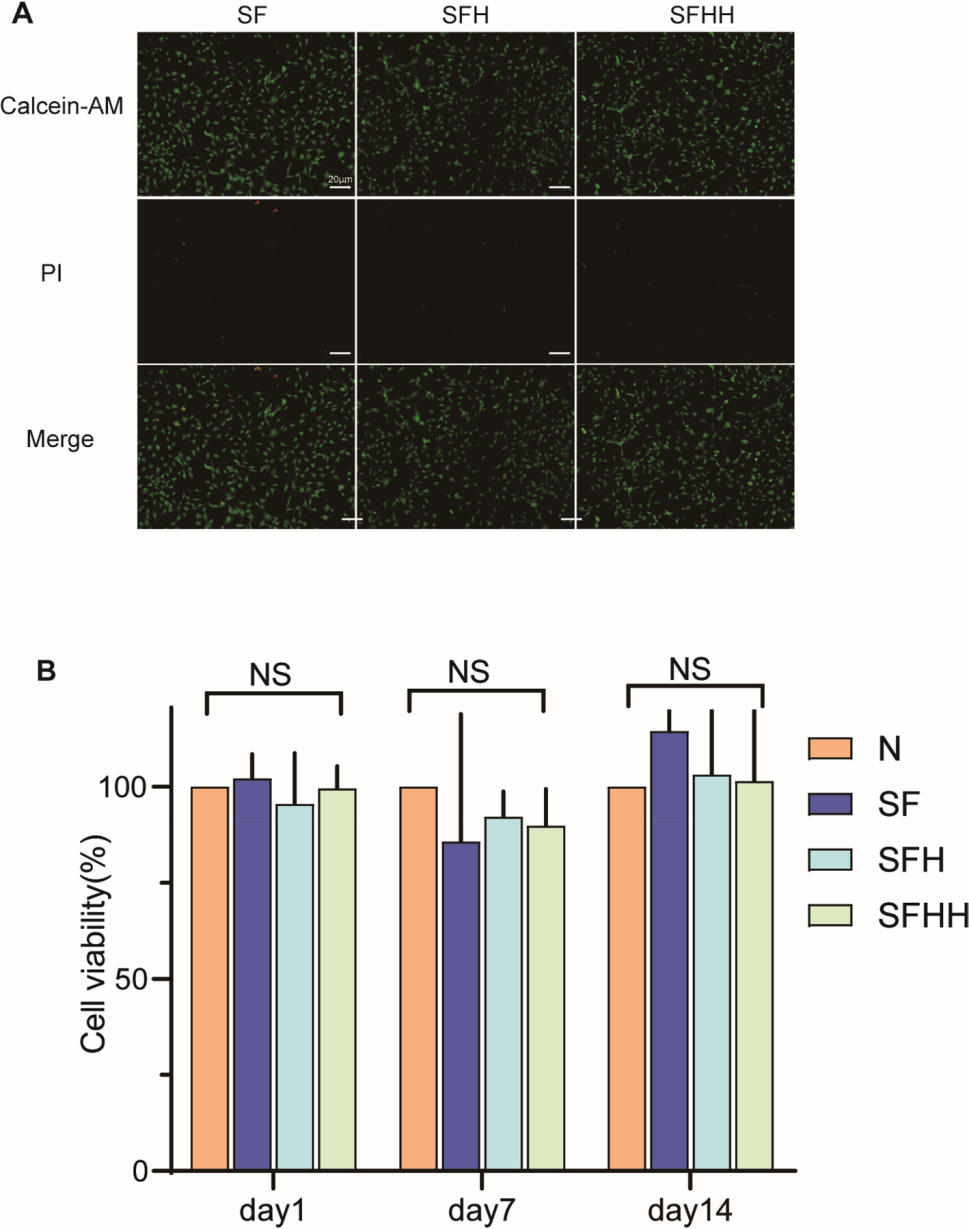
Proliferation evaluation of the BMSCs with nHA/MASF scaffolds. **A** Living/dead staining on different scaffolds of the nHA/MASF scaffolds. **B** CCK-8 assay evaluating the cytotoxic effects after 1–7 days coincubation with different media containing 10% of the extracts, N: Without scaffold, SF: nHA/MASF = 1:0, SFH: nHA/MASF = 1:05, SFHH: nHA/MASF = 1:1,* p* < 0.05, *n* ≥ 3

Live/dead staining showed that most of the inoculated BMSCs were alive, with very few cells dying (Fig. 3A). CCK-8 test results of 1 day, 4 days and 7 days of culture in scaffold extract showed that there was no significant difference in cell viability among the four groups at each same time node of culture (*P* > 0.05) (Fig. 3B). Cells were all cultured as usual, were not contaminated and had no morphological abnormalities like cell shrinkage and cytoclasis. These data revealed that the nHA/MASF scaffold constructed in this study did not show significant cytotoxicity. And the no significant difference among the four groups at each same time showed the scaffolds did not promote cell proliferation under simple conditions that cells were cultured in α-MEM medium without medium for rat BMSCs osteogenic differentiation.

### The scaffold promotes osteogenic differentiation of rat BMSCs in vitro.

**Fig. 4:**
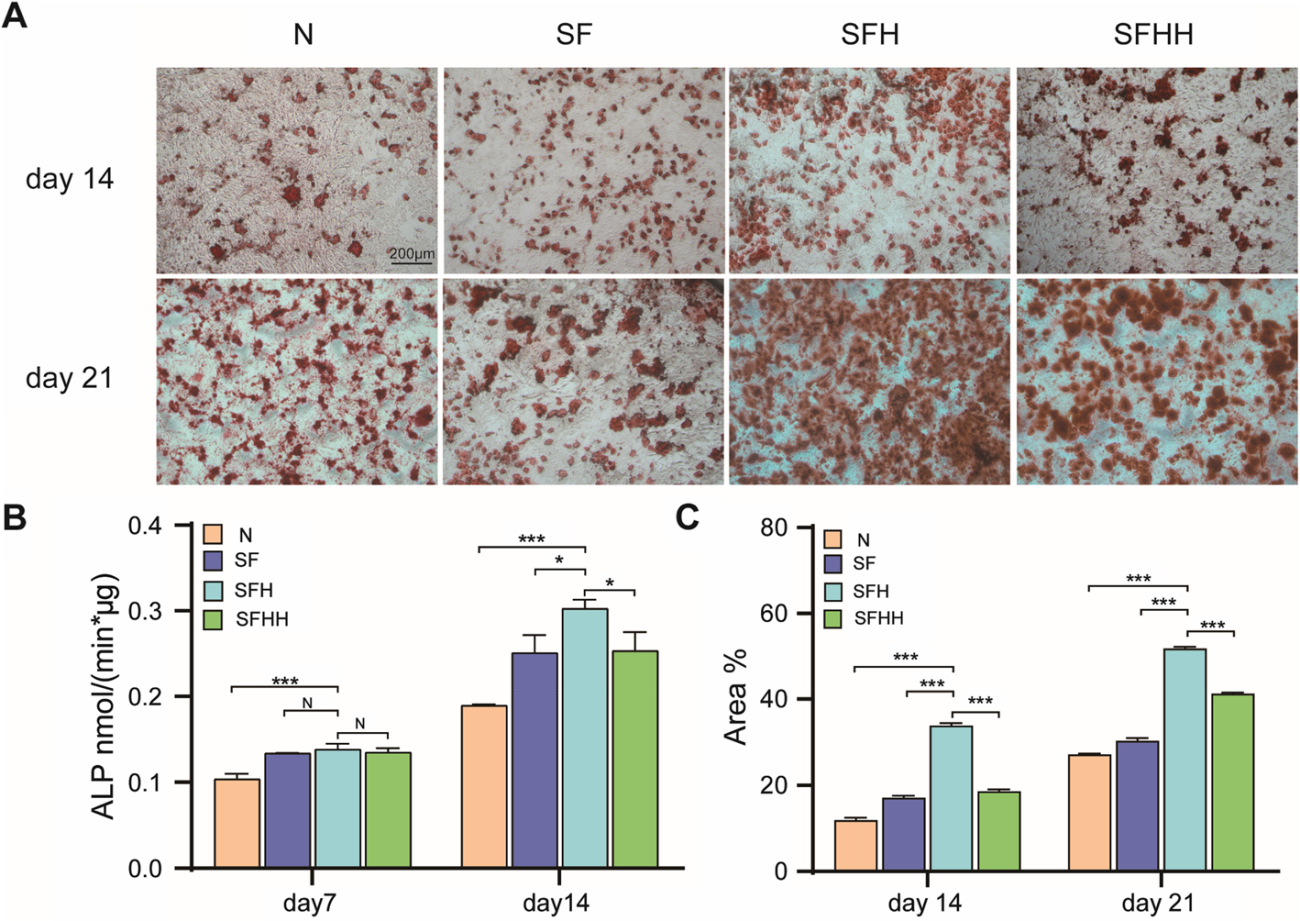
Osteogenic differentiation effects of nHA/MASF scaffolds on BMSCs. **A **Representative photographs of ARS staining on Day 14 and Day 21. **B** Quantitative analysis of ALP staining of BMSCs after being cultured using the conditioned medium of medium containing 10% of the extracts for 7 and 14 days. **C** The area analysis of ARS staining of BMSCs after being cultured using the conditioned medium containing 10% of the extracts for 14 and 21 days. N: Without scaffold, SF: nHA/MASF = 1:0, SFH: nHA/MASF = 1:05, SFHH: nHA/MASF = 1:1,* p* < 0.05, *n* ≥ 3

The efficiency of the mineralization stage was analyzed by using alizarin red S staining as a marker for inorganic calcium, a common feature of bone-like structures. The formation of mineralized nodules was observed under the microscope after a specified time of osteogenic induction (Fig. 4A). The results showed that at Day 21 of induction, cells in the SFH group had more calcium nodules than those in the other groups (Fig. 4A). Then, the area of alizarin red staining was analyzed by Image J (Fig. 4C). Quantitative analysis outcomes revealed that the area of mineralized nodules in group SFH was the largest at 14 and 21 days, and The distinction was significant in statistical terms (*P* < 0.001). Furthermore, on Day 14, the area of mineralized nodules in the SFHH groups was larger than the SF ones (*P* < 0.05). By Day 21, the difference between the two groups increased significantly (*P* < 0.001).

The ALP activity of BMSCs was measured at 7 and 14 days of induction and the results were quantitatively analyzed (Fig. 4B). According to quantitative analysis, after 7 days of culture, the BMSCs in each group showed lower ALP activity, but the group with scaffold extract showed significant differences compared with the blank group. At Day 14, bone marrow MSCs in the SFH group showed higher ALP activity, with a significant difference as compared to the other ones (*P* < 0.05). The findings indicated that the scaffold constructed in this work have osteogenic ability, and the best osteogenic performance was achieved when the ratio of MASF to nHA was SFH.

**Fig. 5:**
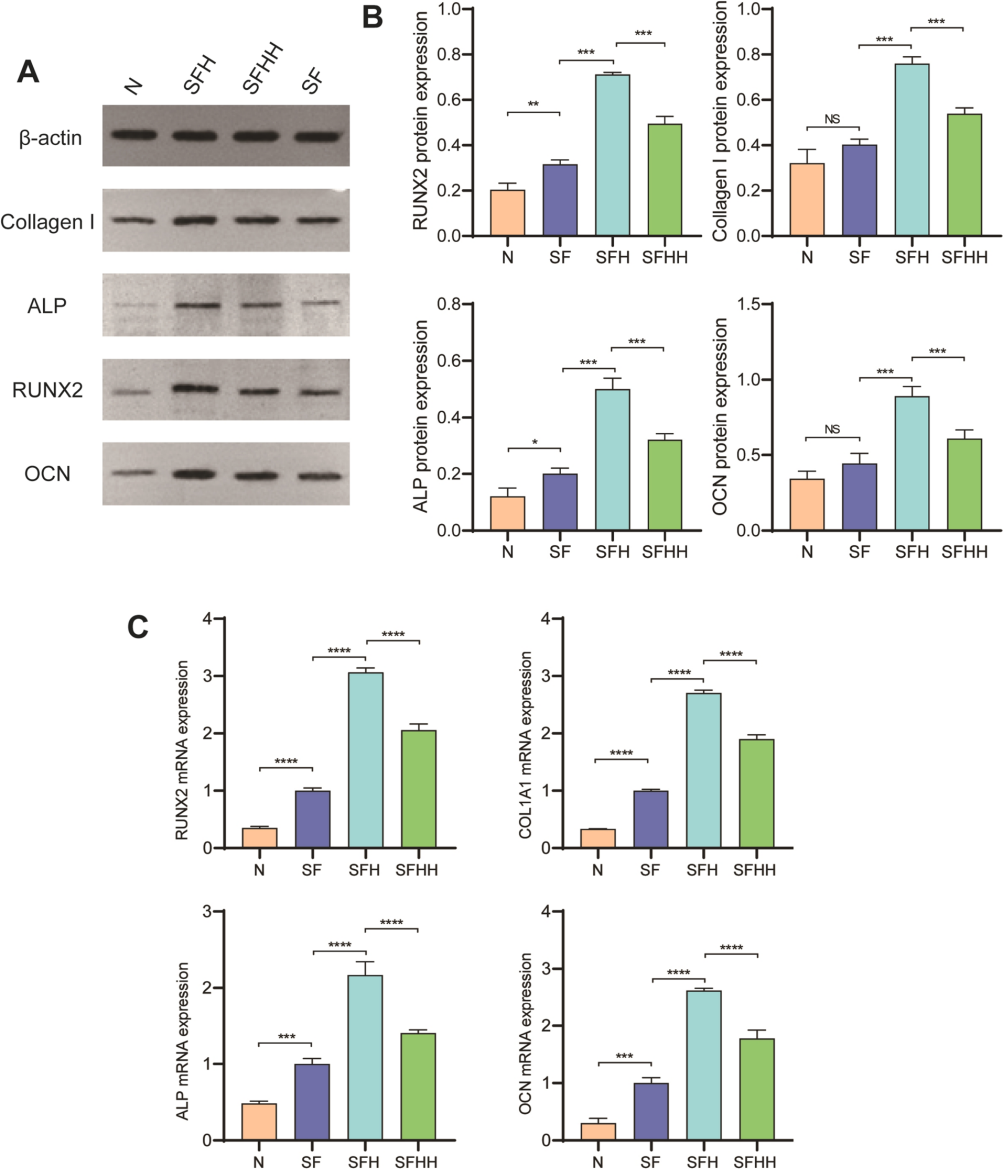
Osteogenic differentiation effects of the nHA/MASF scaffolds on BMSCs. **A**&**B** Western blot analysis results for the protein expression of collagen type I, ALP, Runx2 and OCN on Day 14. **C** Real-time PCR results for the mRNA expression of collagen type I, ALP, Runx2 and OCN on Day 14. N: Without scaffold, SF: nHA/MASF = 1:0, SFH: nHA/MASF = 1:05, SFHH: nHA/MASF = 1:1,* p* < 0.05, *n* ≥ 3

To clarify the effect of different scaffolds on the expression of osteogenic differentiation-related genes and proteins in BMSCs, we studied several marker genes and proteins which that are essential during osteogenesis. The results showed that the bone differentiation related genes Runx2, osteocalcin (OCN), collagen type I and alkaline phosphatase (ALP) were significantly upregulated in SFH after 14 days of culture in OIC medium. However, cells cultured in ordinary OIC medium showed relatively low gene expression and were used as blank controls (Fig. 5C). The corresponding osteogenic differentiation related proteins, including OCN, ALP, Runx2 and type I collagen, produced by bone marrow mesenchymal stem cells were also detected (Fig. 5A, B). Similarly, among all bone-related protein deposits, the SFH group was markedly superior to the other groups.

These results suggest that adding an appropriate amount of nHA to MASF can enhance the osteogenic ability of the scaffold. Compared with the other groups, the SFH group drastically improved the mineralized matrix generation, ALP activity and osteogenic ability of BMSCs.

### Scaffolds promote bone regeneration in vivo

**Fig. 6:**
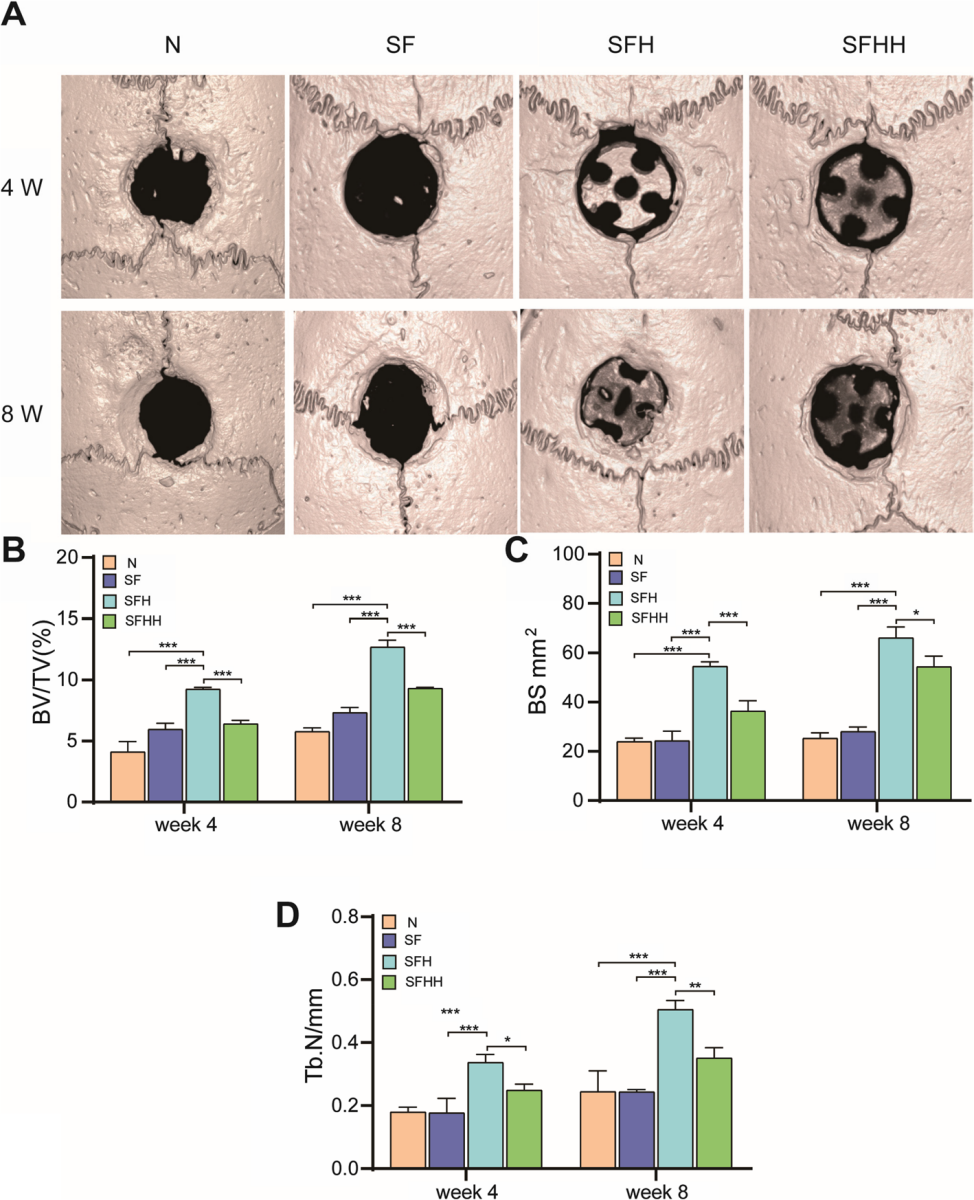
In vivo osteogenesis performance of nHA/MASF scaffolds. **A** Coronal views of characteristic micro-CT reconstruction images from various groups at weeks 4 and 8 following surgery. The date of (**B**) BV/TV: Bone volume/ total volume, (**C**) BS: Bone surface, and (**D**) Tb. N: Trabecular numberin the defect area displaying micro-architectural parameters of newly-formed bone tissue in distinct groups at 4 and 8 weeks post-surgery N: Without scaffold, SF: nHA/MASF = 1:0, SFH: nHA/MASF = 1:05, SFHH: nHA/MASF = 1:1, *p* < 0.05, *n* ≥ 3

On the basis of the results obtained in the in vitro study, this paper further investigated the osteogenic function of the 3D-printed nanohydroxyapatite/methylacrylylated silk fibroin scaffold and SD rat skull defect model. During the operation, the thick lamellar stent was tremendously consistent with the round defect. During the study after stent implantation, the SD rats survived perfectly without infection or death. As revealed by micro-CT, in the controls without scaffolding, there was little inward growth of new bone. For the stent group, with the augmentation of healing time, it can be observed that new bone grows into the stent, and the SFH group has the best radiologic performance during all time intervals. The micro-CT imaging depict a more accurate morphology of new bone integration after stent implantation (Fig. 6A). Through micro-CT, this study first obtained two-dimensional and three-dimensional images and microarchitecture parameters and then quantitatively analyzed the shape and quantity of new bone formation in defects. Most of the borehole defects in the comparison group stayed blank during the research, demonstrating that the self-healing ability of SD rats skull defects was poor. For the scaffold group, the new bone grew from the edge around the defect to the center of the scaffold. Apart from that, this study also observed that there were a multitude of scattered bony tissues within the scaffold, signaling that the composite scaffold not only played a supporting role in the bone defect, but also promoted the adhesion of nearby tissues. Moreover, the multilayer scaffold structure also induced osteocytes to grow inward. Over time, in all groups, more bone was observed to grow into the stent in the SFH groups at 4 and 8 weeks. Regenerated BV/TV ratio, bone Tb. N and bon BS were ascertained by utilizing the micro-CT results obtained. As revealed by bone histomorphometry, the newly formed bone tissue in each group was more than that at 4 weeks after the stent was implanted in mice for 8 weeks. In SD rate bone defect implants, the BV/TV, Tb. N and BS of the SFH group were remarkably higher than those of the other groups at two time points (Fig. 6B, C, D). The results also further verified the influence of micro-CT on the images.

**Fig. 7:**
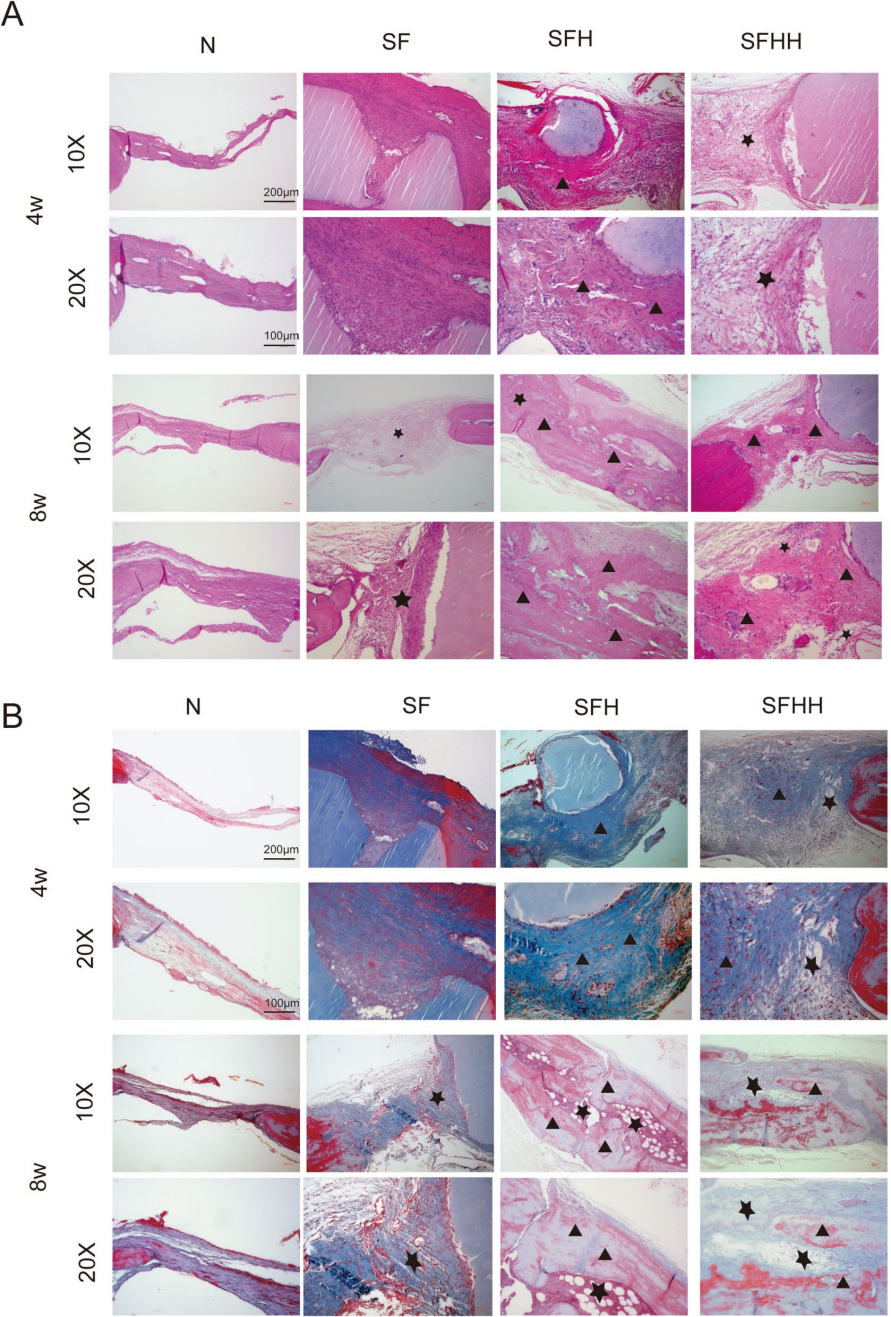
Histological staining of craniums with large cranial defects implanted with nHA/MASF scaffolds. **A** Hematoxylin–eosin (HE) and(**B**) Masson’s trichrome staining (MST) of SD rat skull defects with nHA/MASF scaffolds for 4 and 8 weeks. Black triangles mark newly mineralized bone. Black pentagrams indicate newly connective tissue. N: Without scaffold, SF: nHA/MASF = 1:0, SFH: nHA/MASF = 1:05, SFHH: nHA/MASF = 1:1, *p* < 0.05, *n* ≥ 3

At 4 and 8 weeks after the operation, bone regeneration was further confirmed by HE staining and Masson trichrome staining. As depicted in Fig. 7A, the HE staining diagram, in comparison with the control group without scaffold, the scaffold group had significant new bone formation. In addition, the SFH group had more obvious new bone filling. The regenerated collagen in the blue and mature bone tissues was marked with Masson staining (Fig. 7B) for histological analysis. When compared to the control group, the scaffold group filled in more new collagen at the defect site, indicating that the scaffold promoted the formation of bone matrix. In the SFH group, a large number of new bones were formed, bone trabeculae were arranged neatly, and some bone trabeculae formed woven bone. As indicated by the above results, the SFH group in the scaffold constructed in this study had a better osteogenic effect in vivo.

## Discussion

The artificial manufacture of hydroxyapatite is mainly produced in the form of micron and nanometer particles [35]. However, many recent studies have shown that nanohydroxyapatite has better bone conductivity because its size is similar to natural hydroxyapatite found in bone [36, 37]. Studies have shown that compared with micron hydroxyapatite, nanohydroxyapatite is better at improving protein adsorption and osteoblast adhesion. It also plays an important role in mineral formation [38, 39]. Cai et al. [40] showed that compared with traditional hydroxyapatite, nanohydroxyapatite improved the cytophilicity of bone marrow mesenchymal stem cells, especially 20 nm particles. Yang et al. [41] found that the addition of nanohydroxyapatite into nanofibers can indeed enhance the differentiation of dental pulp stem cells into odontoblast-like phenotypes in vivo and in vitro, and the size of nanohydroxyapatite has a particularly significant effect on odontoblast-like phenotypes. In this study, 20 nm hydroxyapatite particles were used, and the results also showed that the scaffold containing nanohydroxyapatite particles could promote the differentiation of BMSCs and bone defect repair in vivo. Multiple studies [42–44] have demonstrated that the presence of micropores and rough surfaces enhances the specific surface area of scaffolds, promoting the adsorption of proteins and the release and redeposition of calcium ions. These factors significantly contribute to the osteoinductive capabilities of the scaffolds. Nanostructured scaffolds offer architectural support to preserve the transport space for cells and functional factors. What is more, modifying the appearance of a stent to have a rougher surface and properly sized pores could impact the sensitivity of stem cells to mechanical forces and influence their development, including their ability to multiply, adhere, and differentiate into bone cells.

Biomaterials constructed by silk fibroin are currently one of the most widely studied methods [45]. Silk fibroin is soluble in water in its α -helix and random curl forms. Depending on the storage temperature, pH and concentration of the silk solution, the solubility can last for days or even weeks [46]. Silk fibroin requires the transformation of α -helical and random curly conformations into highly stable β-folds to provide good resistance to dissolution, heat, and enzyme degradation [47]. The structure of silk fibroin can be fully adjusted during spinning or regeneration to obtain different secondary structures to control the properties of the material. For example, by forcing silk proteins out of the nozzle, an appropriately altered fiber microstructure can be obtained with significantly high fiber toughness [48]. If optimized for other forms of filament-based materials, these changes may match their load-carrying properties to the target tissue. The structure and surface morphology of silk fibroin formed by the process can also affect biodegradation [49], cell interaction [50] and drug release kinetics [51]. For example, temperature-controlled steam annealing is used to modify the crystallinity to adjust the thermal, mechanical, and biodegradation properties of the filaments [49]. On the other hand, in recent years, the use of di-tyrosine crosslinking has gained popularity as an approach for forming silk hydrogels. Silk hydrogels produced using di-tyrosine crosslinking exhibit both elastomeric and transparent properties. The diverse range of crosslinking techniques developed thus far enables the utilization of this method in various applications, such as biofabrication, photolithography, in situ crosslinking, and cell encapsulation [23]. Although silk fibroin has been well studied, more research is needed to reveal the structure–property relationship to fully control the material properties. This control will be key to the success of silk as a natural biopolymer for tissue regeneration. In this study, MASF was obtained by modifying silk fibroin with glycidyl methacrylate. The silk fibroin molecule was transformed into a photosensitive biomaterial by introducing double bonds. The modified silk fibroin can be crosslinked and cured with a photocrosslinking agent under UV light, which can alter the water solubility and hardness of silk fibroin protein after curing.

This study was intended to first design a biological composite material containing organic and inorganic substances of HA and silk fibroin and then use it as a filling functional material for bone defects and repair bone regeneration. An nHA/ MASF biological composite scaffold was successfully prepared by photoetching 3D printing. As revealed by the physical and chemical characterization results, the scaffold perfectly preserved the three-dimensional space structure of SF without other obvious chemical impurities. Dissimilar concentrations of HA doping not only change the morphology of the scaffold, but also heighten the surface area of pure silk fibroin, which can better promote cell adhesion. In the past, scaffolds made of silk fibroin and HA were also explored and constructed. Nevertheless, the construction methods used in these studies are different, so the performance and morphology of the scaffold are also sissimilar. Bhumiratana et al. [52] mixed HA into NaCl particles, and then poured the silk fibroin solution onto the mixture. Granular NaCl was used as a porogen to prepare scaffolds with pore diameters between 500 and 600 mm. After the scaffold was formed, it was treated with methanol for 1–2 days to induce β- folding and the bracket was cut into the desired shape. Liu et al. [53] prepared the scaffold by dropping the silk fibroin solution into the HA suspension and freeze-drying. In comparison with the previous study, the stent constructed by 3D printing in this study is more controllable. Moreover, the aperture, size and shape of the stent can be precisely customized to achieve personalized use.

Furthermore, as illustrated in the results of this study, the extract of the scaffold constructed by photoetching 3D printing did not noticeably promote cell proliferation in the cells of each group at each time point of 1 day, 4 days, and 7 days of culture in coculture with cells in vitro. Moreover, as proven by live/head cell staining experimental results, the scaffold has no cytotoxicity and the biological characteristics of composite ideal scaffold. In vivo experiments, according to our observations, all the animals in the trial were able to eat independently within 3 days after the operation. The groups that received the scaffold implants showed good healing of the defect areas after the surgery, and there was no worsening of any inflammation-related adverse reactions in the defect areas. There was no infection or tissue death observed in the areas where the scaffold was implanted. The results from the vivo and live/dead staining as well as the CCK-8 assay consistently showed that the 3D-printed scaffolds exhibited excellent biocompatibility. When a substance is directly implanted into the body, it is important to assess its biocompatibility.

The scaffold also displayed advantageous osteoinductive properties in vitro. Osteogenic activity is a paramount characteristic of biomaterials, which is a necessary process for BMSCs to differentiate into osteoblasts, followed by extracellular matrix mineral deposition and formation of bone tissue [54, 55]. Alizarin red S staining confirmed the deposition of calcium phosphate in the BMSC culture and differentiation system (Fig. 4, A). As illustrated by the results of this test, the combined effect of silk fibroin and nHA components in the composite scaffold promoted the mineral deposition of BMSC differentiation and the mineralization of the cell matrix. Furthermore, when the ratio of silk fibroin to HA was 1:0.5, the scaffold also showed higher ALP activity. ALP is not only the main enzyme in the process of bone mineralization [56], but also the early marker of osteoblast differentiation [57]. To clarify the effect of dissimilar scaffolds on the expression of osteogenic differentiation related genes and proteins in BMSCs, we explored several essential marker genes and proteins in the process of osteogenesis. RUNX2 [58] is a distinct type of transcription factor that controls the production of matrix proteins in osteoblasts. It plays a crucial role in the process of osteogenic differentiation and the development of bone. Type I collagen [59] is a crucial constituent of the bone matrix synthesized by osteoblasts, playing a vital role in osteoblast adhesion, differentiation, and bone matrix production. Furthermore, OCN [60] proteins have a tendency to manifest towards the conclusion of osteogenic differentiation. The results indicated that the expression of the bone differentiation related genes Runx2, OCN, type I collagen and ALP [56] was markedly upregulated after SFH was cultured in OIC medium for 14 days. Moreover, cells cultured in ordinary OIC medium exhibited comparatively low gene expression. Meanwhile, the osteogenic differentiation related proteins produced by bone marrow mesenchymal stem cells were detected, including osteocalcin, alkaline phosphatase, Runx2 and collagen I. Likewise, all bone related protein deposits in the SFH group were noticeably higher than those in the other groups. The results of these in vitro experiments indicated that the scaffolds of SFH have good osteogenic capacity. The following reasons may have contributed to this result:(1) The scaffolds in this study combined the organic material silk fibroin and the inorganic compound HA to obtain satisfactory degradation rates. (2) Nano hydroxyapatite can be degraded by alkaline phosphatase to form ions such as Ca^2+^, PO_4_^3−^, and HPO_4_^2−^, which are then absorbed by the cells for bone repair and reconstruction to form new bones. Additionally, nano hydroxyapatite has the ability to enhance alkaline phosphatase activity [61]. Chang et al. [62] discovered HA/calcium sulfate scaffold with the faster disintegration rate, subsequently it was implanted into a femur bone defect of a rat. Following a 13-week period of implantation, degradation occurred, accompanied by the formation of new bone and angiogenesis. (3) Silk fibroin can be partially degraded, and its main component is cell adhesion-related protein. It not only has no adverse reactions to tissues, but also nourishes cells and repairs, which is beneficial to promote the formation of extracellular matrix [63].

The results also suggested that when the content of HA was excessively high, the osteogenic activity decreased. However, the higher the inorganic HA content is, the more it conforms to the composition of human bone. Nevertheless, due to the difficulty of printing materials and the influence of scaffold decomposition and component exudation, although the inorganic calcium content is higher, osteogenesis is reduced. In addition, studies have illustrated that the uppermost factor affecting biocompatibility is hydrophilicity. By increasing the content of HA in the mixed SF and HA scaffold, the hydrophilicity of the scaffold increased with the reduction in the contact angle, exceeding the optimal contact angle of the scaffold. As a consequence, the content of HA in the scaffold was adjusted to obtain a suitable cell adhesive material [64]. Although SF with polar—OH and—COOH groups has medium affinity for water, its hydrophilicity should be further enhanced to ameliorate biocompatibility by combining it with HA with additional—OH and phosphate groups. These highly hydrophilic groups make the diffusion of water droplets more beneficial, which in turn helps to ameliorate the hydrophilicity. The hydrophilicity of SF/HA decreases continuously with increasing HA content. In the investigated samples, SF/HA with an HA content of approximately 30% shows the highest hydrophilicity [65]. These studies are similar to our findings.

After the scaffold was implanted into SD rats, no death was found, indicating that the scaffold also had favorable biocompatibility in vivo. Through micro-CT, we found that there was more bone formation in the middle of the bone defect at the stent implantation site, and a small amount of bone formation at the edge. Wu et al. constructed a scaffold containing nano MgO hydroxyapatite/silk fiber. In vivo experiments suggested that there was more osteogenesis around the scaffold at the bone defect, which grew from outside to inside. Our results differ from those of the above scholars, which indicates that the scaffold constructed in this study can better induce osteoblasts to adhere to and enter the scaffold. Moreover, the CT results also demonstrated that the rats with a 1:0.5 stent implantation ratio had more osteogenesis. Apart from that, bone tissue measurement results also demonstrated that the ratio of BV/TV the in 1:0.5 group was higher. Moreover, BS and Tb. N were also greater than those in the other groups. Furthermore, as revealed by HE and Masson staining results, more new bone was generated in the 1:0.5 group of mice. As indicated by these research findings, the scaffold constructed in this study has excellent osteogenic properties and is a potential bone biomaterial for bone tissue engineering.

## Conclusions

In this work, we constructed an nHA/MASF biocomposite by photocurable 3D printing. The scaffold has excellent biocompatibility and osteogenic function. An osteogenic microenvironment was constructed in vivo to accelerate the repair of bone defects. The best osteogenic effect was achieved when the mass ratio of nHA and MASF fibroin in the scaffold was 1:0.5. The scaffold can be used as a potential bone defect filling material for bone regeneration (Supplementary Materials 1, 2, 3, 4 and 5).

### Supplementary Information

**Supplementary Material 1.**

**Supplementary Material 2.**

**Supplementary Material 3.**

**Supplementary Material 4.**

**Supplementary Material 5.**

## Data Availability

No datasets were generated or analysed during the current study.
