# Supplementary figures and images for: 3D-printed nanohydroxyapatite/methylacrylylated silk fibroin scaffold for repairing rat skull defects
Source: J Biol Eng. 2024 Mar 21;18:22. doi: 10.1186/s13036-024-00416-5 (PMC10956317; doi:10.1186/s13036-024-00416-5)

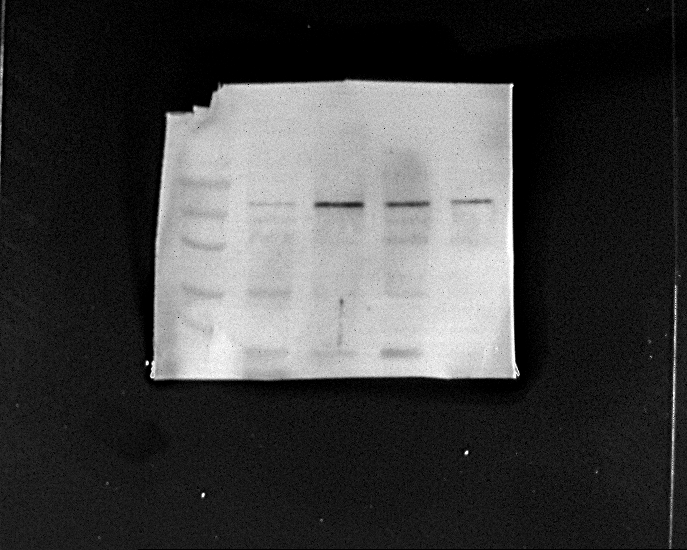

Supplement: Supplementary file 1 — Supplementary Material 1. [file 13036_2024_416_MOESM1_ESM.tif]

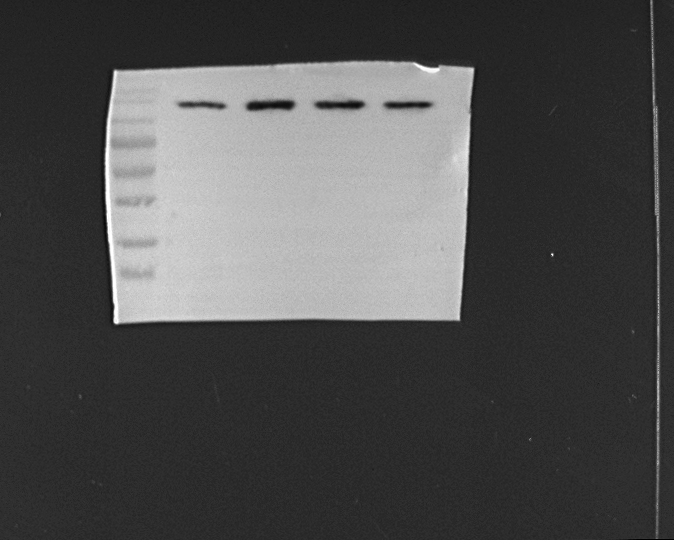

Supplement: Supplementary file 2 — Supplementary Material 2. [file 13036_2024_416_MOESM2_ESM.tif]

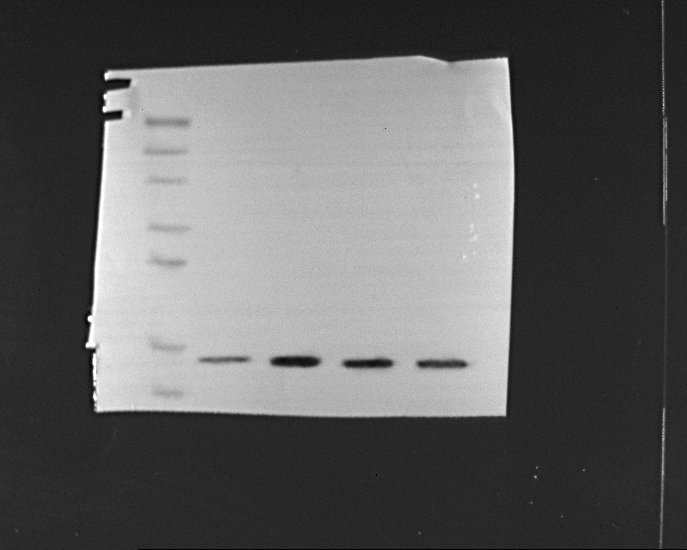

Supplement: Supplementary file 3 — Supplementary Material 3. [file 13036_2024_416_MOESM3_ESM.tif]

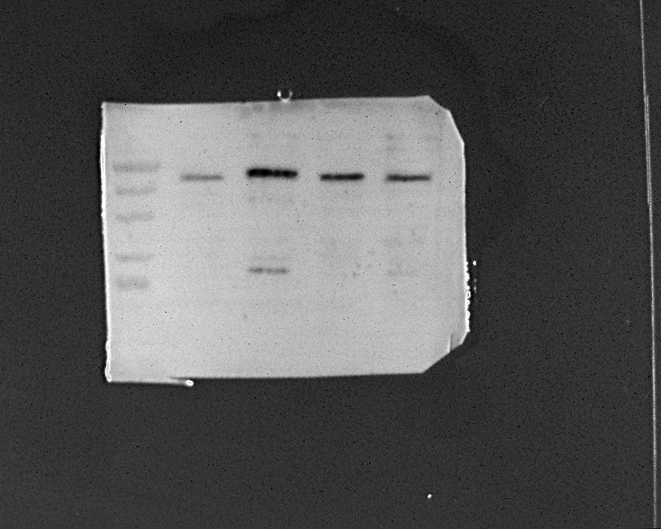

Supplement: Supplementary file 4 — Supplementary Material 4. [file 13036_2024_416_MOESM4_ESM.tif]

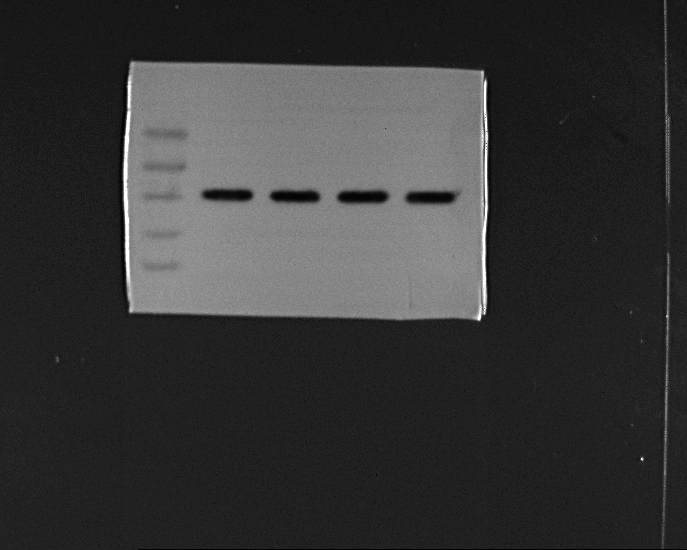

Supplement: Supplementary file 5 — Supplementary Material 5. [file 13036_2024_416_MOESM5_ESM.tif]
